# A novel anti-HER2 antibody GB235 reverses Trastuzumab resistance in HER2-expressing tumor cells *in vitro* and *in vivo*

**DOI:** 10.1038/s41598-020-59818-2

**Published:** 2020-02-19

**Authors:** Mengjun Shu, Hongbin Yan, Chuanying Xu, Yan Wu, Zhaohua Chi, Weihong Nian, Zhuzi He, Jing Xiao, Hongli Wei, Qing Zhou, Joe X. Zhou

**Affiliations:** 1 Key Laboratory of Thin Film and Microfabrication (Ministry of Education), Department of Micro/Nano Electronics, School of Electronic Information and Electrical Engineering, Shanghai Jiao Tong University, Shanghai, 200240 People’s Republic of China; 2 Genor Biopharma Co., Ltd. Building 3, 1690 Zhangheng Rd., Shanghai, 201203 People’s Republic of China; 3 Shanghai Escugen Biotechnology Co., Ltd. 800 Na Xian Rd., Suite 517, Pudong District, Shanghai, 201210 People’s Republic of China

**Keywords:** Cancer therapeutic resistance, Drug development

## Abstract

HER2 overexpression is frequently associated with tumor metastasis and poor prognosis of breast cancer. More evidence indicates that HER3 is involved in HER2-resistant therapies. Combination treatments with two or more different monoclonal antibodies are a promising strategy to overcome resistance to HER2 therapies. We presented a novel fully human HER2-targeted monoclonal antibody, GB235, screened from a phage-display library against the HER2 antigen. GB235 in combination with Trastuzumab overcomes resistance in HER2-positive tumors and results in more sustained inhibition of tumor growth over time. The competition binding assay showed that the epitopes of GB235 do not overlap with those of Pertuzumab and Trastuzumab on HER2. Further HER2 mutagenesis results revealed that the binding epitopes of GB235 were located in the domain III of HER2. The mechanism of action of GB235 in blocking HER2-driven tumors is different from the mechanisms of Trastuzumab or Pertuzumab. GB235 does not affect the heterodimerization of HER2 and HER3, whereas the GB235 combined treatment with Trastuzumab significantly inhibited heregulin-induced HER3 phosphorylation and downstream signaling. Moreover, GB235 in combination with Trastuzumab reversed the resistance to heregulin-induced proliferation in HER2-overexpressing cancer cell lines. GB235 combined with Trastuzumab treatment in xenograft models resulted in improved antitumor activity. Complete tumor suppression was observed in the HER2-positive NCI-N87 xenograft model treated with the combination treatment with GB235 and Trastuzumab. In a Trastuzumab-resistant patient-derived tumor xenograft model GA0060, GB235 plus Trastuzumab reversed the resistance to Trastuzumab monotherapy. Because GB235 showed a different working mechanism with Pertuzumab and Trastuzumab, these agents can be considered complementary therapy against HER2 overexpression tumors.

## Introduction

HER2, also known as ErbB2, is a member of the HER receptor family, which also consists of EGFR (ErbB1), HER3 (ErbB3), and HER4 (ErbB4) receptors^1^. Overexpression of HER2 is considered a biomarker of malignant tumors with a poor prognosis and is presented in roughly 20~30% of breast cancer patients^2^. HER2 is also overexpressed in approximately 20% of gastroesophageal junction and gastric cancers with a poor prognosis^3^. Trastuzumab is a humanized anti-HER2 antibody and it is the first HER2-targeted therapeutic monoclonal antibody approved by the United States Food and Drug Administration (FDA) in 1998 for the therapy of metastatic HER2-positive breast cancer and in 2010 for the therapy of HER2-positive metastatic gastroesophageal junction and gastric cancer^4,5^. Trastuzumab inhibits HER2 phosphorylation and consequently inhibits downstream signaling pathways^6^. More and more studies indicate that Trastuzumab inhibits ligand-independent HER3/HER2 interactions rather than blocking HER2 signaling^7^. However, not all patients with HER2 overexpression benefit from Trastuzumab therapy because of the initial or acquired resistance^8^. The precise mechanism of resistance to Trastuzumab is still unclear. More evidence indicates that the HER2-HER3 heterodimer^9^ and its consequent downstream signaling play a crucial role in the tumor resistance and metastasis of HER2-positive cancer^10^. Many metastatic HER2-amplified cancers that either do not respond to, or are eventually resistant to Trastuzumab often recover the phospho-HER3 and PI3K-Akt-mTOR downstream signaling^11^.

Pertuzumab, a recombinant humanized monoclonal anti-HER2 antibody, sterically blocks HER2 heterodimerization with other HER receptors, especially HER3, and was approved by the FDA in 2012 for breast cancer therapy^12^. Pertuzumab and Trastuzumab bind to distinguishing epitopes on HER2, and the combination treatment inhibits HER2/HER3 signaling more effectively^13^. Trastuzumab combined with Pertuzumab and docetaxel was approved as a first-line therapy for HER2-positive metastatic breast cancer^14^. HER2-targeted bispecific antibodies in clinical development have demonstrated significant antitumor activity for the Trastuzumab-resistant malignant cancers in preclinical studies^15,16^. MM-111, a bispecific antibody that specifically targets the HER2/HER3 heterodimer, blocks heregulin binding and inhibits downstream signaling pathways. Trastuzumab alone had no effect on heregulin-induced paclitaxel resistance, whereas MM-111 in combination with Trastuzumab displayed significantly greater activity than either agent alone^17^. Ertumaxomab, a bispecific anti-CD3 × anti-HER2 antibody, targets HER2 on cancer cells and CD3 on T cells simultaneously. The trifunctional bispecific antibody can evoke immune immunocytotoxicity towards HER2-overexpressing cancer cells and exhibited antitumor activity^18^. Despite remarkable improvement in HER2-targeted therapy, a large number of patients face the risk of recurrence. Therefore, efforts in developing more efficient HER2 inhibitors to increase overall survival rates are necessary.

Here, we describe the characteristics and effects of a fully human antibody, GB235, binding to domain III of HER2 and recognizing different epitopes on HER2 than the ones recognized by Trastuzumab or Pertuzumab. The combination treatment of GB235 and Trastuzumab reversed the resistance to heregulin-driven proliferation in BT-474 and NCI-N87 HER2-overexpressing tumor cells. GB235 in combination with Trastuzumab demonstrated significant tumor growth inhibition in the NCI-N87 HER2-overexpressing xenograft model. Tumor growth was significantly inhibited by combined treatment with GB235 and Trastuzumab in a Trastuzumab-resistant patient-derived tumor xenograft (PDX) GA0060 compared to Trastuzumab alone. Therefore, GB235 in combination with Trastuzumab might be a potential therapy against HER2-positive tumors.

## Results

### Characterization of the novel anti-HER2 antibody

**Figure 1:**
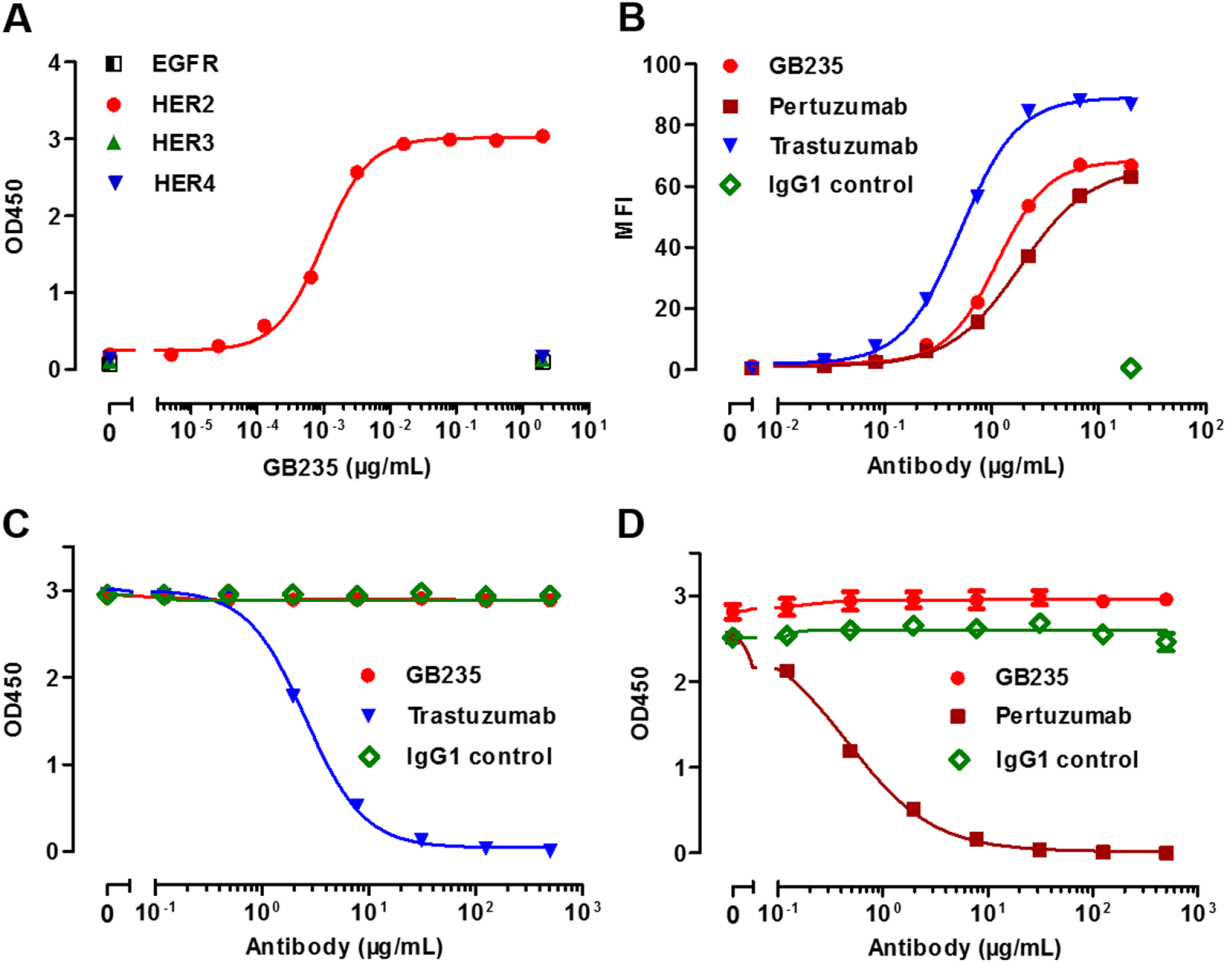
The immunoreactivity of the fully human antibody GB235 against HER2. (**A**) GB235 specifically bound to soluble human HER2 ECD but not to the other human EGFR family receptors. (**B**) Flow cytometry analysis of GB235 binding to HER2-overexpressing BT-474 cells. MFI: mean fluorescence intensity. GB235 was evaluated for its activity to compete with biotinylated Trastuzumab (**C**) and biotinylated Pertuzumab. (**D**) The results are representative of three different experiments and expressed as the mean ± SEM.

To identify a HER2 therapeutic antibody, we screened HER2-targeted scFvs using phage display technology against the human HER2 extracellular domain (HER2 ECD) antigen. The full IgG1 molecules were generated from the top twenty scFv colonies based on their affinity ranking to HER2 ECD. The superior candidate antibody showing the best inhibition against the heregulin-induced phosphorylation of HER3 was designated as GB235. The binding activity of GB235 for the antigen was evaluated in the recombinant human HER2 ECD proteins or HER2-overexpressing BT-474 cells. The binding specificity of GB235 to soluble human HER2 receptor was determined by an ELISA. The results showed that GB235 specifically bound to human HER2 ECD in a concentration-dependent manner, without binding to other EGFR family receptors (Fig. 1A). The cell binding assay was investigated with flow cytometry, the GB235 specifically bound to BT-474 cells, which is consistent with the result of Trastuzumab and Pertuzumab (Fig. 1B). The competition of binding activity was performed by an ELISA. The data demonstrated that GB235 does not compete with biotinylated Trastuzumab (Fig. 1C) or biotinylated Pertuzumab (Fig. 1D), suggesting that GB235 does not bind to the same epitopes recognized by Trastuzumab or Pertuzumab on human HER2.

The affinity of GB235 for its antigen was evaluated by surface plasmon resonance. The kinetic parameters of the Biacore X 100 system gave similar *K*_D_ values for human HER2 and cynomolgus HER2 (Table 1). The dissociation coefficient (*K*_D_) for human and cynomolgus HER2 was 1.69 nM and 2.18 nM, respectively.

**Table 1:** The binding kinetics of GB235 determined by biacore.

	k_a_ (1/Ms)	k_d_ (1/s)	*K*_D_ (M)	Rmax (RU)	Chi² (RU²)
Human HER2	3.70 × 10^5^	6.25 × 10^−4^	1.69 × 10^−9^	49.11	1.8
Cynomolgus HER2	3.53 × 10^5^	7.68 × 10^−4^	2.18 × 10^−9^	51.76	5.12

### Antiproliferative effects of GB235 and HER2 family status

**Figure 2:**
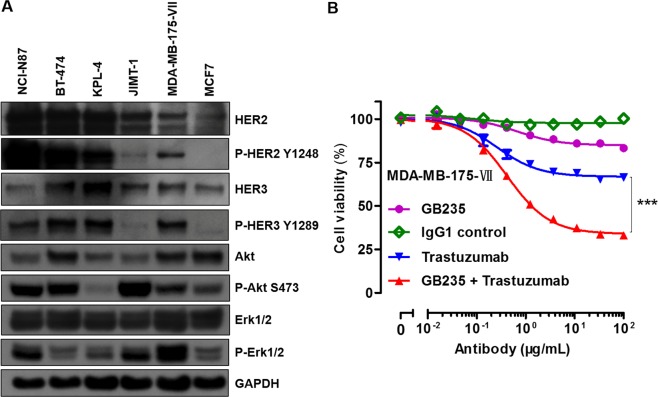
(**A**) Expression of HER family protein levels and downstream signal activity of cell lines. Cell lysates were harvested for western blot analysis of HER2, HER3 and downstream signaling mediators of Akt and Erk1/2. GAPDH as a loading control. (**B**) Treatment of MDA-MB-175-VII cells with GB235 in combination with Trastuzumab for five days resulted in an enhanced antiproliferative effect. The results are representative of three experiments. The data are expressed as the mean ± SEM. ***p < 0.001.

To additionally evaluate whether HER family status conveys sensitivity to HER2 neutralizing antibody GB235, we studied the HER2, HER3 levels and downstream signal activity of a panel of cancer cell lines. As shown in the Fig. 2A, the amount of HER2 was overexpressed in NCI-N87, BT-474, KPL-4 cells and moderately expressed in JIMT-1 and MDA-MB-175-VII cells, but was significantly lower in MCF7 cells. HER2 was heavily tyrosine phosphorylated in NCI-N87, BT-474 and KPL-4 cells. Although JIMT-1 moderately expressed HER2, we could detect only very low levels of P-HER2, which is consistent with a previous report^19^. Most of the cancer cell lines analyzed also expressed elevated levels of HER3. The constitutive expression of P-HER3 levels were higher in examined cancer cell lines, except in JIMT-1 and MCF7 cells. Total Akt and Erk1/2 proteins were expressed in all of the examined cell lines at various levels. The levels of P-Akt were higher in NCI-N87, BT-474 and JIMT-1 cells, but were lower in KPL-4 cells. The Erk1/2 pathway was more active in NCI-N87, JIMT-1 and MDA-MB-175-VII cells compared with that of BT-474, KPL-4 and MCF7 cells.

We further investigate the activation of HER3-HER2 signaling and its potential role in mediating resistance to Trastuzumab monotherapy. Previous studies indicate that the growth of MDA-MB-175-VII cells is dependent on autocrine growth factor of γ-heregulin through the HER3-HER2 signaling^20^. The combination of Pertuzumab with Trastuzumab-DM1 showed enhanced antiproliferative activity on MDA-MB-175-VII cells compared with either drug alone^21^. We found that either Trastuzumab or GB235 showed a partial antiproliferative activity against MDA-MB-175-VII cells, whereas the GB235 combined with Trastuzumab showed enhanced antiproliferative activity with a greater extent than each agent alone (*p* < 0.001) (Fig. 2B).

### GB235 retains efficacy in the presence of heregulin-α in BT-474 cancer cells with acquired resistance to Trastuzumab

**Figure 3:**
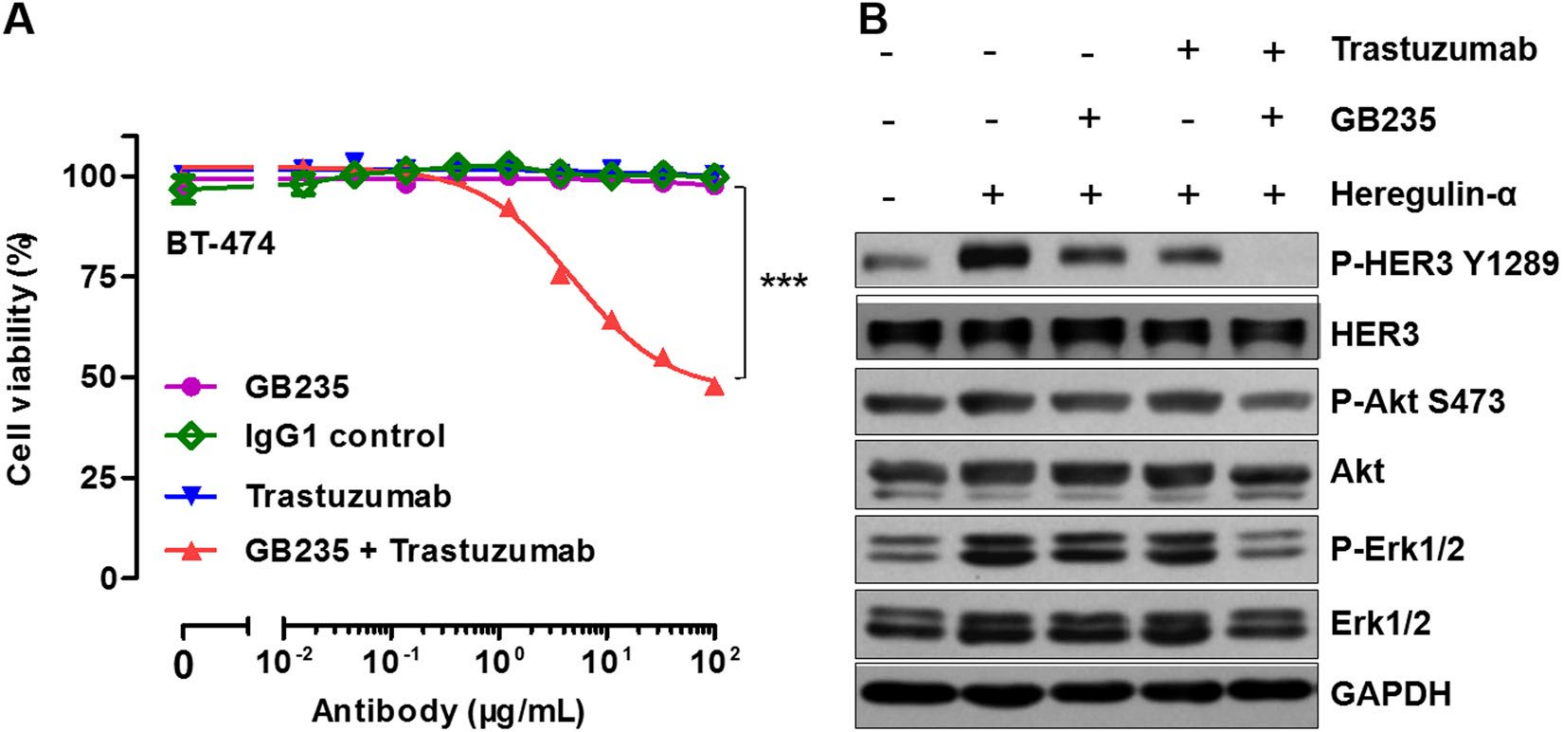
GB235 in combination with Trastuzumab reversed resistance induced by heregulin-α in BT-474 cells by the restraining heregulin-α/HER3 pathway. (**A**) In the presence of exogenous heregulin-α, the BT-474 cells were resistant to Trastuzumab. With the addition of GB235 to Trastuzumab, BT-474 cells recovered sensitivity to Trastuzumab. The data are expressed as the mean ± SEM of three independent experiments. ***p < 0.001. (**B**) Serum-starved BT-474 cells pretreated with GB235 (20 µg/mL), Trastuzumab (20 µg/mL) alone or in combination, then stimulated by heregulin-α, and immunoblotted.

To examine the efficacy of GB235 in combined treatment with Trastuzumab *in vitro*, we analyzed cell growth inhibition against heregulin-α-induced proliferation in HER2-overexpressing BT-474 cells^22^. The induced cell proliferation by heregulin-α in BT-474 cells was not inhibited by Trastuzumab treatment alone, while the combination treatment with GB235 and Trastuzumab inhibited heregulin-α-induced cell proliferation significantly (*p* < 0.001) (Fig. 3A). In contrast, no combination efficacy was observed in BT-474 cells treated in the absence of heregulin-α (data not shown).

To explore whether GB235 was able to inhibit heregulin-α-mediated HER3 activation and downstream signaling, BT-474 cells were treated with GB235 alone or in combination with Trastuzumab followed by exposure to heregulin-α. Treatment with GB235 or Trastuzumab alone to some extent inhibited the phosphorylation of HER3 and downstream signaling effectors of Erk1/2, only the combination treatment of GB235 and Trastuzumab resulted in completely attenuation of HER3 phosphorylation and downstream signaling of Erk1/2 (Fig. 3B).

### The reverse effect of GB235 in combination treatment with Trastuzumab in NCI-N87 cancer cells

**Figure 4:**
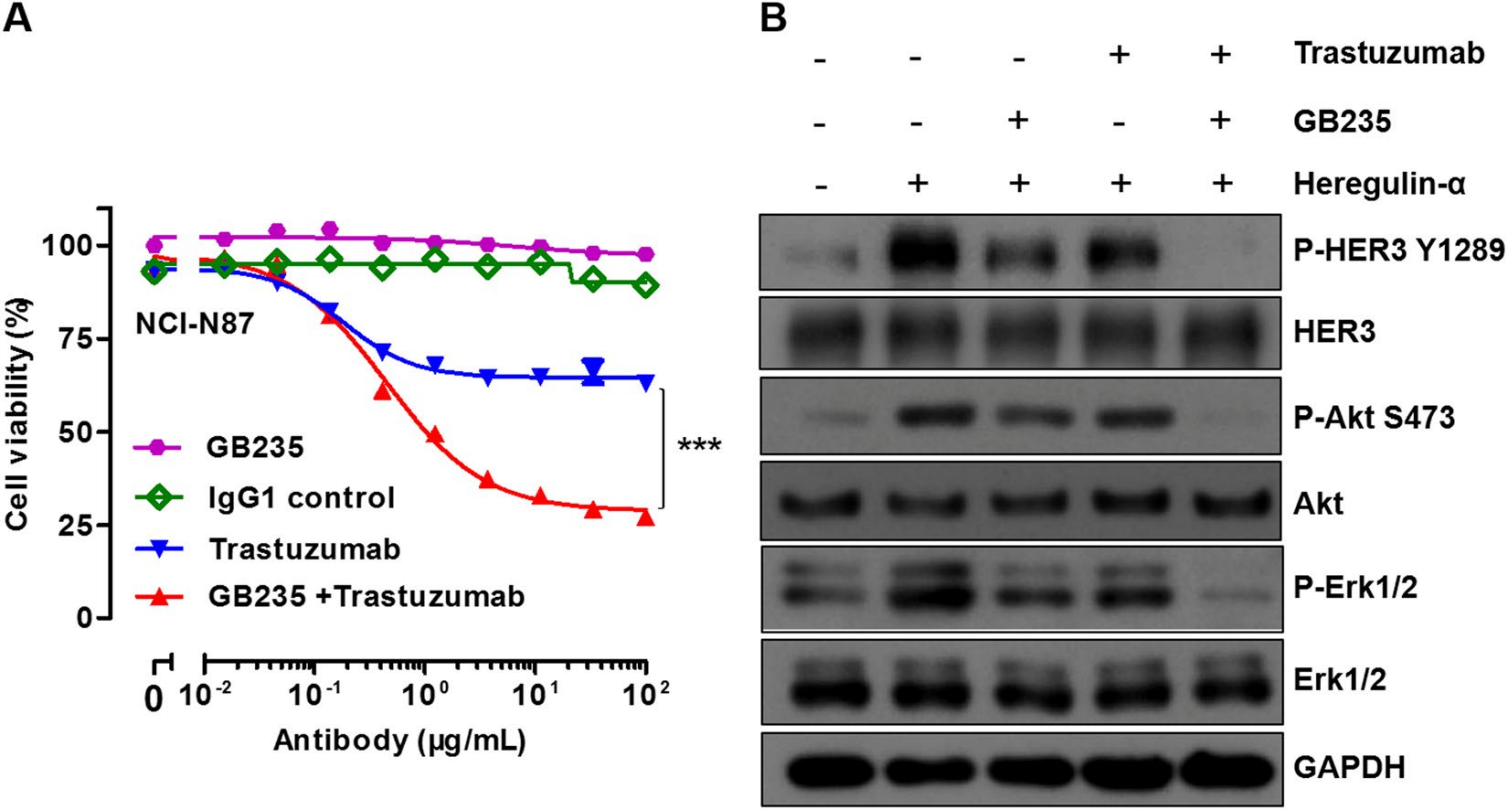
Effect of GB235 alone or in combination treatment with Trastuzumab in NCI-N87 cells. (**A**) Enhanced proliferation inhibition with combination treatment of GB235 and Trastuzumab was shown in NCI-N87 cells in the presence of heregulin-α. (**B**) Western blot analysis of HER3 and downstream signaling pathway activation upon GB235 (20 µg/mL) or Trastuzumab (20 µg/mL) alone, or the two antibodies in combination treatment followed by heregulin-α stimulation.

The antiproliferative activity of GB235 was examined in Trastuzumab-resistant gastric cancer cells in the presence of heregulin-α. HER2-overexpressing NCI-N87 gastric cancer cells are resistant to Trastuzumab monotherapy *in vitro* and *in vivo*^23^. Under heregulin-α-induced conditions, Trastuzumab alone showed a minor proliferation inhibition in the NCI-N87 cells. However, GB235 combined with Trastuzumab treatment displayed superior *in vitro* proliferation inhibitory activity compared with either of the antibody (*p* < 0.001) (Fig. 4A).

Next, we studied the inhibitory effects of GB235 or Trastuzumab alone, or the two antibodies in combination, on HER3 signaling activation in NCI-N87 cells. Upon heregulin-α stimulation, the phosphorylation of HER3 and downstream signaling mediators of Akt and Erk1/2 were assessed after the treatment with the antibodies in NCI-N87 cells. The results indicated that the GB235 or Trastuzumab alone had a partial effect on phosphorylation of HER3, Akt and Erk1/2, whereas GB235 in combination with Trastuzumab significantly decreased P-HER3, P-Akt and P-Erk1/2 (Fig. 4B). The heterodimer of HER2/HER3 and downstream signaling play a pivotal role in tumorigenesis of NCI-N87 cells^24^.

### Heterodimerization inhibition assay and antibody-dependent cell-mediated cytotoxicity assay

**Figure 5:**
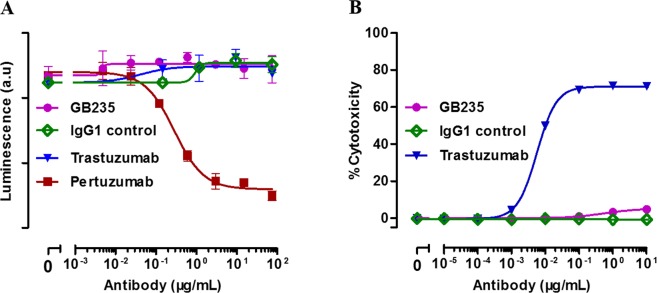
(**A**) Effect of GB235 on the heterodimerization inhibition. (**B**) The ADCC effect of GB235 and Trastuzumab on JIMT-1 target cells. The data are expressed as the mean ± SEM of three independent experiments.

The next aim of this study was to explore whether GB235 binding to HER2 ECD prevents the heterodimerization of HER2/HER3. U2OS (HER2-HER3) used in this study is a commercialized engineering cell line (DiscoverX, Fremont, USA), which stably expresses the extracellular domains of the HER2 receptor fused to the ProLink and HER3 fused to EA. The high signal to noise ratio makes the assay applicable to the detection of ligand-induced dimerization of HER2-HER3 receptors in the therapeutic neutralizing antibody studies^25^. As shown in Fig. 5A, the dose-response curve of Pertuzumab showed an inhibitory effect of heterodimerization induced by heregulin-1-β1. However, neither GB235 nor Trastuzumab directly inhibited the heterodimerization of HER2 and HER3.

JIMT-1, derived from a breast cancer patient shows HER2 amplification and resistance to Trastuzumab monotherapy. In previous publications, Trastuzumab evoked antibody-dependent cell-mediated cytotoxicity (ADCC) on JIMT-1 cancer cells with dose-dependent cell death reaching 70 to 85% killing^26^. The ADCC activity induced by Trastuzumab was equally strong against JIMT-1or SKBR-3 cancer cells^27^. The JIMT-1 cells were used in our study to determine the antibody-dependent cell-mediated cytotoxicity. In contrast with Trastuzumab, GB235 had no ADCC effect on the JIMT-1 target cells (Fig. 5B).

### GB235 targeted epitopes located in the domain III of HER2

**Figure 6:**
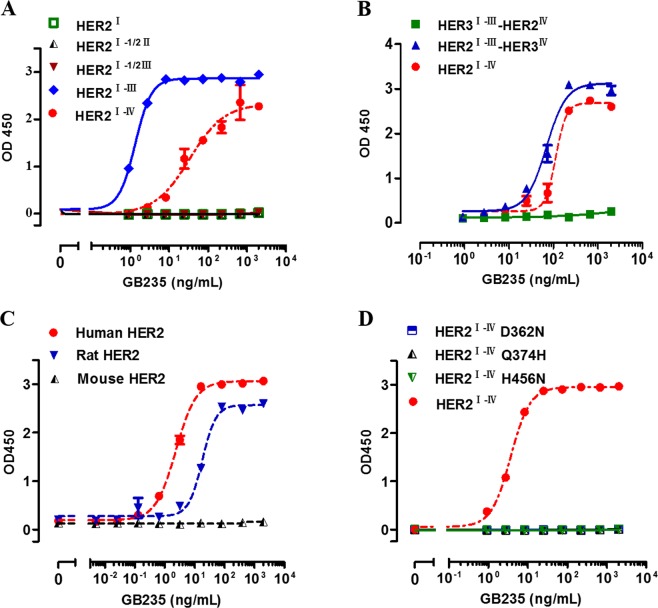
(**A**) Evaluation of the binding activity of the truncated mutants of HER2 ECD d^I^, HER2 ECD d^I-1/2II^, HER2 ECD d^I-1/2III^ and HER2 ECD d^I-III^ with GB235 as determined by ELISA. (**B**) Binding ability of chimeric HER2^I-III^-HER3^IV^ and HER3^I-III^-HER2^IV^ with GB235 as evaluated by ELISA. (**C**) The cross-reactivity of GB235 toward mouse, rat or human HER2 was evaluated by ELISA. (**D**) Binding activity of human HER2 ECD point mutants (D362N, Q374H and H456N) to GB235 as determined by ELISA. The data are expressed as the mean ± SEM of three independent experiments.

To explore the mechanism by which GB235 inhibits HER2/HER3 signaling, efforts were made to identify the epitopes of GB235 on human HER2. HER2 ECD is composed of four domains (I, II, III and IV), associated with flexible regions. The truncated mutants of HER2 ECD d^I^, HER2 ECD d^I-1/2II^ and HER2 ECD d^I-1/2III^ lost binding activity to GB235. In contrast, the HER2 ECD d^I-III^ showed reactivity toward GB235 (Fig. 6A). Furthermore, the binding ability of the chimeric proteins of HER2 and HER3 to GB235 was evaluated. The result showed that HER2^I-III^-HER3^IV^ was sufficient to mimic full-length human HER2 ECD d^I-IV^ in the association with GB235. The HER3^I-III^-HER2^IV^ was unable to bind to GB235 (Fig. 6B). The species cross-reactivity of GB235 was determined by assessing the binding activity to HER2 ECD proteins of three different species. GB235 specifically bound to human and rat HER2 ECD, but did not cross-react with the mouse HER2 ECD in the ELISA (Fig. 6C). Moreover, it was observed that 362D, 374Q, 395D, 456 H are phylogenetically conserved in human and rat HER2 ECD d^III^, but not in mouse HER2 ECD d^III^. The residues are conserved in the human and rat HER2 but not in the mouse HER2, thus explaining the lack of cross-reaction with mouse HER2. The binding of the full-length human HER2 ECD d^I-IV^ to GB235 was abolished in site-directed mutants of human HER2 (D362N, Q374H and H456N) (Fig. 6D). The residues 362D, 374Q and 456 H in the human HER2 ECD d^III^ were found to be critical for the binding of GB235.

### GB235 combined with Trastuzumab effectively inhibits tumor growth in xenograft models

**Figure 7:**
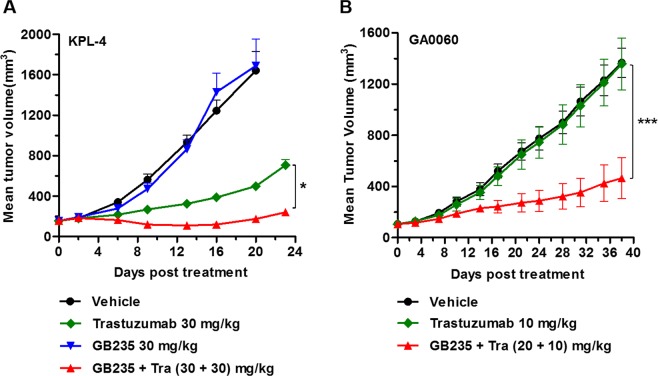
(**A**) Effect of GB235 and Trastuzumab on KPL-4 xenograft. SCID mice bearing KPL-4 tumors were randomized into four groups when tumor volumes reached 100∼200 mm^3^ (n = 6 mice/each group). The KPL-4 xenograft was intraperitoneally injected with GB235 (30 mg/kg), Trastuzumab (30 mg/kg), combination treatment of GB235 (30 mg/kg) and Trastuzumab (30 mg/kg), or negative control. *p < 0.05. (**B**) For the patient-derived tumor xenograft GA0060 model, when the tumor volume was approximately 100 mm^3^ (n = 5 mice/group), the nude mice were treated with Trastuzumab (10 mg/kg) or in combination with GB235 (10 + 20 mg/kg). Tra: Trastuzumab. Data are presented as the mean tumor volume ± SEM. ***p < 0.001.

The ability of the GB235 to block cell signaling and suppress cell proliferation *in vitro* supported the analysis of its therapeutic efficacy *in vivo*. We investigated the *in vivo* tumor growth activity of GB235 in combination with Trastuzumab in breast cancer and gastric cancer cells. KPL-4 xenograft tumors (Breast cancer) were strongly positive for HER2 staining^28^. Combination treatment with Trastuzumab and Pertuzumab dramatically enhanced the antitumor effect compared with each agent alone in KPL-4 xenograft model^29^. As shown in Fig. 7A, the administration of Trastuzumab alone (30 mg/kg) in mice bearing KPL-4 breast cancer xenograft induced significant tumor growth inhibition, while the GB235 alone (30 mg/kg) had no effect on the tumor growth. However, the anticancer activity of GB235 in combination with Trastuzumab in KPL-4 xenograft was significantly higher than Trastuzumab as a single agent (*p* < 0.05). Consistent with the results showing that the addition of GB235 to Trastuzumab is more effective at suppressing cancer cell proliferation *in vitro*, the combination of GB235 and Trastuzumab is also trended toward enhanced tumor growth inhibition *in vivo*.

Next, to evaluate whether combined GB235 and Trastuzumab could be an effective therapeutic strategy to overcome Trastuzumab resistance *in vivo*, we assessed the efficacy of GB235 and Trastuzumab in two other Trastuzumab-resistant human gastric cancer xenografts. For mice bearing of gastric patient-derived tumor xenograft, the PDX model GA0060 showed no response to Trastuzumab monotherapy^30^. In contrast, tumor growth was significantly suppressed in nude mice treated with combinatorial treatment with GB235 plus Trastuzumab in comparison to mice treated with Trastuzumab alone (Fig. 7B) (*p* < 0.001).

**Figure 8:**
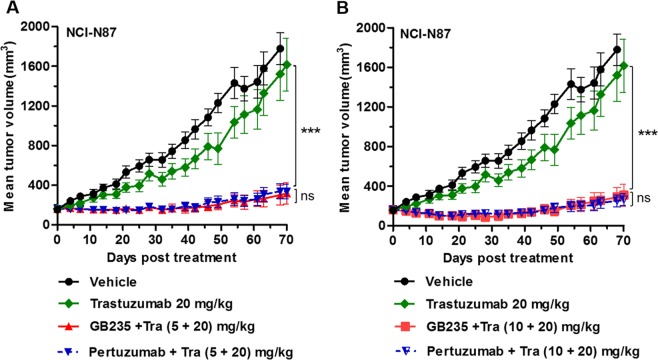
GB235 in combination treatment with Trastuzumab inhibited tumor growth in the NCI-N87 gastric xenograft tumor model. BALB/c nude mice bearing NCI-N87 tumors were randomized into treatment and control groups when tumor volumes reached 100∼150 mm^3^ (n = 10 mice/group) and intraperitoneally treated with: (**A**) Trastuzumab alone (20 mg/kg), combination treatment of GB235 and Trastuzumab (5 + 20 mg/kg), (**B**) GB235 and Trastuzumab (10 + 20 mg/kg). The same dose ratios of combination treatment with Pertuzumab and Trastuzumab as control. Tumor volume is expressed as the mean ± SEM. ***p < 0.001.

To further explore the potential of GB235 to overcome Trastuzumab resistance *in vivo*, we evaluated the antitumor activity in an additional Trastuzumab-resistant xenograft model. In the HER2-positive NCI-N87 xenograft, Trastuzumab monotherapy displayed poor ability in tumor growth inhibition^31^. To this end, we assess the efficacy of the combinational treatment of GB235 and Trastuzumab in the NCI-N87 xenograft model, as inhibition of HER3 in the HER2-positive gastric cancers exhibits a promising efficacy^32^. Notably, GB235 combined with Trastuzumab exhibited significant antitumor activity in NCI-N87 xenograft, whereas Trastuzumab alone displayed only partial effect (*p* < 0.001). Both dose ratios of GB235 and Trastuzumab (5 + 20 mg/kg, and 10 + 20 mg/kg) significantly inhibited tumor growth by 84.1% and 83.6%, respectively (Fig. 8A,B). A head-to-head comparison between the GB235 and Pertuzumab, combination treatment with GB235 and Trastuzumab inhibited tumor growth to a similar level as the treatment with the Pertuzumab plus Trastuzumab. Collectively, the results demonstrate that the addition of GB235 to Trastuzumab treatment sensitizes Trastuzumab-resistant cancer cells to Trastuzumab.

## Discussion

Despite the fact that the prognosis of HER2 overexpressing metastatic breast cancer has been significantly improved with Trastuzumab therapy, more efficient therapies are necessary for relapsing or resistant cases. Heregulin rescues the cancer cells from the inhibitory effects of HER2 kinase antagonists through HER3 activation^33^. Moreover, the HER3 mediates resistance to the inhibitory effect of HER2, EGFR, and PI3K kinase inhibitors via HER3 up-regulation and activation^34^. Herein, we show that a fully human anti-HER2 antibody, GB235, in combination with Trastuzumab overcomes resistance to Trastuzumab monotherapy *in vitro* and *in vivo*. Either the autocrine or exogenous heregulin-induced cell proliferation was inhibited by the combined treatment of GB235 and Trastuzumab. The analysis of the HER2 and HER3 signaling pathway indicated that GB235 inhibits cancer cells by reducing the activation of the HER3 receptor and the downstream pathway effectors of Akt and Erk1/2. In line with the *in vitro* results, the combination therapy of GB235 and Trastuzumab demonstrated a strong and enduring antitumor effect in HER2-amplified xenograft tumors.

The mechanism of action of GB235 is different from that of Trastuzumab or Pertuzumab. Binding analysis of GB235 with HER2 mutants indicated that GB235 bound to nonoverlapping epitopes on domain III of HER2. Trastuzumab binds to domain IV of HER2 which inhibits the homodimerization between HER2 dimers^35^. Pertuzumab binds to domain II of HER2 and interferes with the heterodimerization between HER2 and the other HER family receptors^36^. Further studies were performed to improve the understanding of the mechanism of action of GB235. Trastuzumab displayed significant ADCC activity in HER2-amplified JIMT-1 cells, whereas GB235 had no such effect. Neither GB235 nor Trastuzumab inhibited the heterodimerization process.

Domain III of HER2 was reported to be involved in the function of HER2 and HER3 interaction^37^. Deletion of the key region (451–466) in domain III of HER2 ECD suppressed the transactivation of receptors and disrupted the constitutive conformation in the dimerization loop, which may lead to the trafficking behavior of intracellular kinase^38^. The asymmetric dimers between intracellular kinases of HER2/HER3 participate in the activation of signaling, as the kinase domain of HER3 serves as an allosteric activator of HER2 kinase^39,40^. Conformation changes were detected in the HER2 activation loop during dimerization interactions with HER3, which were stabilized each other in the heterodimer^41,42^. More reports have indicated that Trastuzumab disrupts noncanonical HER2-HER3 interactions that result in HER3 inactivation^43^. We hypothesize that GB235, once bound to domain III of HER2 ECD, locks the HER2 ECD into an inactive conformation. The combination of GB235 and Trastuzumab is more likely to influence the rearrangement of HER2/HER3, potentially augmenting the steric hindrance effect on the activation of the intracellular kinase.

In summary, we reported a novel fully human monoclonal antibody against HER2, GB235, which bound to domain III of HER2 ECD and displayed potential efficacy inhibiting HER3-mediated signaling in heregulin-induced Trastuzumab resistant cancer cells. This combined therapy of the two antibodies demonstrated superior antitumor effect in the relevant xenograft mouse models than Trastuzumab alone. GB235 in combination with Trastuzumab presents a novel approach for overcoming Trastuzumab-resistant breast and gastric cancers. The unique potential of GB235 provides an alternative or complementary therapeutic strategy to the standard of care for HER2-positive cancers.

## Materials and Methods

### Antibodies and reagents

Trastuzumab and Pertuzumab were purchased from Roche Pharmaceutical Ltd. GB221 was developed as a potential Trastuzumab biosimilar, and is currently in Phase III clinical trial (NMPA Clinical approval number 2013L01513). The extensive side-by-side analysis of GB221 and Trastuzumab showed highly similar physicochemical properties and functional characterization. Both GB221 and Trastuzumab used in this report are collectively referred to as Trastuzumab. IgG1 isotype control was obtained from Crown Bioscience Inc. (Taicang, China). Heregulin-α and heregulin-1-β1 were purchased from R&D Systems Inc. (Minneapolis, USA). The PathHunter assay kit was purchased from DiscoveRx (Fremont, USA). The P-HER3 (Y1289), HER3, P-HER2(Y1248), HER2, Akt, P-Akt (S473), Erk1/2, P-Erk1/2, GAPDH rabbit monoclonal antibodies and Radio Immunoprecipitation Assay buffer (RIPA) were purchased from Cell Signaling Technology Inc (Boston, USA). All antibodies were used according to the manufacturer’s recommended antibody dilutions. Phosphotase inhibitor cocktail and nitrocellulose membrane were obtained from Merck (Darmstadt, Germany). Both CytoTox 96^®^ Non-Radioactive Cytotoxicity Assay kits and CellTiter 96^®^ Aqueous One Solution (MTS) Cell Proliferation Assay kits were purchased from Promega Corporation (Wisconsin, USA).

### Cells and cell culture

MDA-MB-175-VII cell line was purchased from American Type Culture Collection (Maryland,USA). BT-474, MCF7 and NCI-N87 cell lines were obtained from the Cell Bank of the Chinese Academy of Sciences (Shanghai, China). The KPL-4 cell line was provided by Nanjing Cobioer Co., Ltd. (Nanjing, China). JIMT-1 was obtained from the German Collection of Microorganisms and Cell Cultures GmbH (Braunschweig, Germany). Cell lines were incubated at 37 °C under 5% CO_2_ and cultured in Roswell Park Memorial Institute-1640 (RPMI-1640) or Dulbecco’s Modified Eagle Medium (DMEM) supplemented with 10% Fetal Bovine Serum (FBS). The NK-92MI-CD16a cell line was purchased from Huabo Biopharm Co., Ltd. (Shanghai, China) and was maintained in α-Minimum Eagle’s Medium (α-MEM) supplemented with 12.5% FBS, 12.5% Horse Serum, 0.1 mM 2-mercaptoethanol, 0.5 mg/mL of G418, 0.02 mM folic acid, and 0.2 mM inositolat 37 °C under 5% CO_2_. The identity of human cell lines was verified by short tandem repeat DNA typing.

### Selection of a single-chain fragment variable specific for the human HER2 extracellular domain

A fully human single-chain fragment variable (scFv) phage display library containing 7 × 10^10^ individual clones was used for the selection of lead-scFv clones. The phage clones with a high binding activity to human HER2 were selected with phage ELISA. ScFv phage suspension was added into 96-well immuno plates captured with recombinant human HER2 extracellular domain (ECD)-Fc fusion protein and thoroughly washed to remove the nonspecific phage absorption. With four rounds of panning, the phages were eluted with a low pH glycine elution buffer and neutralized with Tris-base and amplified through *E. coli* XL1-blue infection. The scFv phage clones expressed in the bacteria were then purified and characterized. Positive phage clones were analyzed to identify clones with unique DNA sequences.

### Construction and expression of the recombinant full-length IgG1 antibody

The scFv antibodies were converted to full-length IgG1 with an overlapping PCR and determined for their biologic activity. The full-length IgG1 heavy chain was obtained with an overlapping PCR method using the synthesized signal peptide, the variable region of the phage clone heavy chain and the constant region of the synthesized IgG1 heavy chain. The light chain was also obtained with the same strategy. The light and heavy chain of the full-length IgG1 was cloned into an expression vector and transfected into Chinese hamster ovary (CHO) cells with electroporation. Stable cell lines were obtained through incubation in the presence of G418. The recombinant antibody in the serum-free culture medium was purified through Protein G affinity chromatography.

### Immunoassays

The binding ability of GB235 to receptors was determined by Enzyme Linked Immunosorbent Assay (ELISA). Nunc Maxisorp 96-well (flat-bottom) plates were coated with 1 µg/mL of HER family receptors in Phosphate Buffer Saline overnight at 4 °C. Nonspecific binding of antibodies was blocked with 2% Bovine Serum Albumin (BSA) for an hour at room temperature and washed three times with Phosphate Buffer Saline/0.1% Tween-20 (1 × PBST). One hundred microliters of goat Anti-Human IgG Fc-fragment-specific conjugated with Horseradish Peroxidase (1:10000) was added to the 96-well plates and incubated for an hour at room temperature. After washing, the 3,3′,5,5′-Tetramethylbenzidine substrate (TMB) was added to develop color and the absorbance was measured at 450 nm using a SpectraMax M5 plate reader (Molecular Devices, San Jose, USA).

Competition of the binding ability assay was performed. Either biotinylated Trastuzumab or biotinylated Pertuzumab (10 ng/mL) was pre-incubated with GB235 for an hour at 37 °C before adding to the wells of ELISA plate pre-coated with HER2 ECD recombinant protein. After washing with PBST, 100 µL of Streptavidin-Horseradish Peroxidase was added to each well and incubated for an hour at room temperature. Finally, the absorbance of color development was read by SpectraMax M5 plate reader (Molecular Devices, San Jose, USA) at wavelength of 450 nm. The data analysis was performed by GraphPad Prism (GraphPad Software) using a nonlinear, variable slope model.

### Binding to HER2 positive BT-474 cells

A total of 1 × 10^6^ BT-474 cells were added with dilutions of GB235, incubated for an hour at 4 °C in 100 µL of Phosphate Buffer Saline/1% Fetal Bovine Serum (PBS/1% FBS). Either Pertuzumab or Trastuzumab was used as positive control. The BT-474 cells were washed three times in cold PBS/1% FBS buffer and incubated with Fluorescein-conjugated goat anti-human IgG-Fc secondary antibody (1/200 dilution) for 45 min at 4 °C in PBS/1% FBS buffer. The cells were washed with three times, and analyzed using a Beckman Coulter FC500 Cytometer (Beckman, Kraemer Boulevard, USA). A total of 1 × 10^4^ cells was counted for each sample. The binding affinity was analyzed with GraphPad Prism (GraphPad Software).

### Surface plasmon resonance analysis

For binding kinetic studies, the binding of GB235 to HER2 was determined by surface plasmon resonance using a Biacore X 100 (GE Healthcare, Chicago, USA). Carboxyl groups on the chip were activated by N-hydroxysuccinimide and N-ethyl-N-(dimethylaminopropyl) carbodiimide and deactivated with Ethanolamine. GB235 was immobilized on the surface of a sensor chip CM5 via an anti-human IgG Fc antibody using amine coupling. Either human HER2 ECD or monkey HER2 ECD in HBS-EP + solution (GE Healthcare, Chicago, USA) was injected at various concentrations (1.56∼200 nM) over the flow cell. The interaction was determined at a flow rate of 5 μL/min. Data analysis was conducted with a 1:1 Langmuir fit model using BIAevaluation software.

### *In vitro* cell proliferation assays

The effect of GB235 on tumor cell viability was assessed with the CellTiter 96^®^ Aqueous One Solution Cell Proliferation Assay kit (MTS). Cells were plated into 96-well plates (5000 cells per well for BT-474; 20000 cells per well for NCI-N87 and MDA-MB-175-VII cell lines) in complete medium and incubated overnight at 37 °C in a humidified atmosphere of 5% CO_2_. The cell culture medium was replaced by fresh cell medium with serial dilutions of GB235, Trastuzumab alone or in combination (ranging between 0.015 and 100 µg/mL). For the heregulin-induced proliferation assay, cells were precultured overnight in complete medium and treated with antibodies for 2 h before the addition of heregulin-α (final concentration 100 ng/mL). Cells were incubated for three or five days. Cell viability was determined by MTS following the manufacturer’s instructions. The absorbance of plate wells was recorded at 450 nm using a SpectraMax M5 plate reader (Molecular Devices, San Jose, USA).

### Immunoblot analysis

For profiling of cancer cell panels, the cells were cultured in complete medium and lysed with RIPA buffer containing phosphotase inhibitor cocktail and dithiothreitol. For inhibitor treatments, BT-474 and NCI-N87 cells were starved in RPMI-1640 supplemented with 0.1% Fetal Bovine Serum (FBS) for 16 h. Cells were treated with serial dilutions of GB235 alone or in combination with Trastuzumab for 3.5 h at 37 °C/5% CO_2_. Heregulin-α (final concentration 100 ng/mL) was added to cells and stimulated for 10 min. After washing with cold PBS, cells were lysed with 1 × NuPAGE^®^ LDS Sample Buffer plus dithiothreitol (Invitrogen, USA). Cell lysates were electrophoresed on 10% SDS-PAGE gels, and transferred onto nitrocellulose membrane. The membrane was blocked with 5% milk powder for an hour at room temperature before incubation with primary antibodies (Rabbit monoclonal antibodies against P-HER3(Y1289), HER3, P-HER2(Y1248), Akt, P-Akt (S473), Erk1/2, P-Erk1/2 and mouse monoclonal antibodies against HER2, GAPDH, respectively) at 4 °C for 16 h. The membrane washed three times with 1 × PBST and incubated with Peroxidase-AffiniPure^®^ Donkey Anti-Rabbit IgG secondary antibody (1/10000 dilution) or Peroxidase-AffiniPure^®^ Goat Anti-Mouse IgG secondary antibody (1/10000 dilution) at room temperature for another hour. After washing with 1 × PBST, the membrane was incubated with ECL and exposed to an X-ray film.

### Heterodimerization inhibition assay of HER2/HER3

U2OS cells expressing HER2 and HER3 receptors with C-terminal probe peptides were placed into a 96-well tissue culture treated plate and incubated at 37 °C, in 5% CO_2_ for 24 h^44^. GB235, Trastuzumab, Pertuzumab and human IgG1 isotype control were then prepared in cell plating reagent at a three-fold dilution and added to the wells of the plate. The plate was incubated for 60 min at 37 °C. Next, heregulin-1-β1 (final concentration 20 ng/mL) was added to all cells and the plate was incubated for 3 h at 37 °C. Then, detection reagent was added and incubated for 60 min at room temperature. Luminescence was read with an integration of 500 milliseconds using synergy 2 microplate reader (Bio-Tek, Vermont, USA). The luminescence intensity was plotted against the concentration of the antibodies.

### Antibody-dependent cell-mediated cytotoxicity

Either GB235 or Trastuzumab was diluted and each solution was added to a 96-well round plate. Target cells of JIMT-1 and effector cells of NK-92MI-CD16a were mixed at a ratio of 1:5 and incubated at 37 °C and 5% CO_2_ for 4 h. Centrifuge plate at 250 × g for 5 min, 50 μL of supernatant was transferred to a new 96-well flat-bottom plate. Next, 50 μL of CytoTox 96^®^ Reagent (Promega, Madison, USA) was added to the plate and incubated for 30 min at room temperature. Assays were stopped by adding stop solution and the absorbance at 490 nm was recorded by a SpectraMax M5 plate reader (Molecular Devices, San Jose, USA). The following formula was used to analysis the percentage of cytotoxicity: Cytotoxicity % = (Experimental − Effector cells basal − Target cells basal)/(Maximal target cells − Target cells basal) × 100.

### Epitope mapping

HER2 truncated mutants (HER2 ECD d^I^, HER2 ECD d^I-1/2II^, HER2 ECD d^I-1/2III^, HER2 ECD d^I-III^), chimeric receptors of HER2 and HER3 (HER2^I-III^-HER3^IV^, HER3^I-III^-HER2^IV^) and HER2 site-directed mutants (D362N, Q374H, H456N) were created through PCR. All mutants and wild-type proteins in the study were tagged at the C-terminus with a Myc and 6–His sequence. The normalized proteins were used in the binding ELISA. The supernatant of mutants was added to 96-well plates precoated with 1 µg/mL of anti-His antibody, followed by incubation at 37 °C for an hour. The TMB substrate was added and the absorbance was determined at 450 nm with a SpectraMax M5 plate reader (Molecular Devices, San Jose, USA).

### *In vivo* tumor studies

Xenograft models were established by inoculation of human cancer cell lines or patient-derived tumor tissue in immune-deficient mice. For the KPL-4 (breast cancer) xenograft study, 9 × 10^6^ of KPL-4 cells were inoculated orthotopically into the right penultimate inguinal mammary fats pad of 6-week-old female NOD/SCID mice. For NCI-N87 (gastric carcinoma) and gastric tumor patient-derived xenograft GA0060 (gastric carcinoma) *in vivo* studies, 2 × 10^6^ of NCI-N87 cells or 30 mm^3^ of GA0060 tissue were inoculated into the right flank of 6-week-old female BALB/c nude mice. When tumors reached 100∼150 mm^3^, tumor-bearing mice were randomized into vehicle and treatment groups. Tumor-bearing mice were intraperitoneally administered GB235, Trastuzumab alone or in combination once a week (or twice a week), while vehicle group received PBS. Tumors were measured twice weekly using calipers and calculated using the following formula: Volume = Length × Width × Width/2. The relative tumor growth inhibition rate (TGI %) was calculated as follows: TGI % = (1 − T_o_/T_c_) × 100 (where T_o_ is the tumor volume change of the antibody-treated group, T_c_ is the tumor volume change of the vehicle group). The procedures of KPL-4 animal study involving the care and use of animals are reviewed and approved by the Institutional Animal Care and Use Committee in GenScript Bio Co., Ltd. The NCI-N87 animal study protocols were approved by the Institutional Animal Care and Use Committee in Shanghai Model Organisms Center, Inc. The GA0060 xenograft model was carried out upon approval of the Institutional Animal Care and Use Committee of the Crown Bioscience Inc. All animal care was in accordance with institution guidelines.

### Statistical analysis

The assays were repeated two or three times and the results are representative of independent experiments. All experiments were performed in accordance with relevant guidelines and regulations. Statistical analysis was performed using GraphPad Prism version 5.0 (GraphPad Software, USA). Data were analyzed with one-way ANOVA for *in vitro* studies. The differences of tumor growth between the groups were analyzed by two-way ANOVA for *in vivo* studies. The values were presented as the mean ± SEM. *p* < 0.05 was considered statistically significant.
